# Self-administration of a novel subcutaneous bradykinin b_2_ receptor antagonist, icatibant, as an effective treatment option in patients with hereditary angioedema

**DOI:** 10.1186/1710-1492-10-S2-A45

**Published:** 2014-12-18

**Authors:** Stephanie Santucci, Hoang Pham, Rachel Harrison, William Yang

**Affiliations:** 1 Allergy and Asthma Research Centre, Ottawa, ON, Canada; 2 University of Ottawa Medical School, Ottawa, ON, Canada

## Background

Hereditary Angioedema (HAE) is a rare disease characterized by recurrent angioedema attacks involving larynx, abdomen, extremities and various body parts. The reactions are by and large self-limited, but potentially, could be fatal. Until recently, the only approved treatment in Canada is an intravenous C1-esterase inhibitor infusion. However, intravenous therapy can be challenging for those who have co-morbid disorders. Icatibant (Firazyr®) —which received approval in Canada in June 2014 — offers administration through subcutaneous delivery. Through a special access program, here we present self-administered icatibant treatment on a female subject with Charcot-Marie-Tooth disease, a rare genetic, neuromuscular disorder, which limits her ability to self-administer intravenous therapy.

## Methods

During each icatibant self-administration event, a diary method was used to collect the following patient-reported outcomes: attack intensity, anatomical location & trigger, number of doses, onset of relief, time elapsed until complete resolution, and adverse reactions.

## Results

From 2012- May 2014, the patient logged a total of 12 events, in which she treated each attack with a single self-administered 30 mg dose of icatibant via subcutaneous injection. She experienced moderate to severe abdominal and peripheral HAE attacks. Onset of relief occurred within 15 – 30 minutes and complete resolution occurred within 4-hours to 5-days. Adverse reactions were mild in severity, transient, and resolved without further intervention. They included local injection site reaction (100%), headache (58%), fatigue (25%), feeling “fuzzy-brained” (25%), and hot flush (8%).

## Conclusion

This case report provides supporting evidence for icatibant as an effective, safe and viable subcutaneous therapeutic alternative to intravenous treatments for patients with HAE.

